# Hospital Gangrene of Diphtheritic Origin—The Relation of Tubal Pregnancy to Pelvic Hematocele—Peritoneal Adhesions

**Published:** 1895-05

**Authors:** Emory Lanphear

**Affiliations:** 2312 Salisbury Street; St. Louis, Mo.


### HOSPITAL GANGRENE OF DIPHTHERITIC ORIGIN—THE RELATION OF TUBAL PREGNANCY TO PELVIC HEMATOCELE—PERITONEAL ADHESIONS.

By EMORY LANPHEAR, M. D., Ph. D., St. Louis, Mo.

### I.—HOSPITAL GANGRENE.

The question as to the relation of diphtheria to hospital gangrene has never been settled, and in these days of antisepsis when traumatic diphtheria or hospital gangrene has almost entirely disappeared there seems to be little possibility of deciding it by actual study of cases. For this reason the history of a patient recently under my care is of unusual interest. Upon a lady of fifty-three years, who had suffered for many years by reason of an absence of the posterior wall of the bladder from a slough following an attempt to form a vagina where there was congenital absence of uterus and vagina, I made a plastic operation to close the vulva, inserting a catheter for continuous drainage. Everything progressed favorably for sixty hours, when I removed the catheter on account of vesical irritation, instructing the nurse to introduce it in a few hours if the urine was not passed naturally. I then saw a case of diphtheria requiring tracheotomy in a part of the city quite distant from my home and office; while there an urgent message came from the first patient, who was suffering intensely from the retention, the nurse having failed upon trying to pass the catheter. I, therefore, hastened to the hospital without going home to change my clothing or thoroughly sterilize my hands, and hastily replaced the catheter. Relief was immediate and as the patient was in excellent condition next day I left to perform an operation several hundred miles away. On my return I found my patient in a very critical condition. About forty-eight hours after the introduction there was a severe rigor followed by a temperature of 104.5 and most profound prostration. A few hours later the wound and contiguous mucous membrane found covered with a grayish white deposit which the experienced nurse at once characterized as "diphtheritic," although unconscious of any possibility of infection. Late the next day I saw the patient and found a typical, dirty-grey, diphtheritic membrane upon the affected surface, with edema; the temperature 97, pulse thready and the patient unconscious from marked toxemia. I at once opened up the wound and cauterized it with pure carbolic acid, and continued the energetic stimulation which had been instituted by the doctor temporarily in charge. From this time onward the course of the disease was characteristically that of hospital gangrene described as the superficial croupous variety. A large greyish-yellow mass of tissue assumed a tow-like appearance and broke down into a gangrenous pulp, which was thrown off after several days. The sub-normal temperature continued for three days and was succeeded by an intermittent fever of an irregular character, the chart showing no record above 100.5, which lasted for eight days, the general condition gradually improving. The subsequent history was that of diphtheria, including a paralysis of the right arm and hand which became marked in the second week of convalescence; the paralysis disappeared within six weeks after the onset of the traumatic diphtheria.

Here was a case presenting the typical symptoms of "hospital gangrene," unquestionably an infection-disease, and undoubtedly of diphtheritic character—the paralysis alone being sufficiently diagnostic. How Rosenbach and Roser, the two authors who contend strongly against the identity of the two diseases, would explain the conditions I am at a loss to know—possibly that the trouble was not "hospital gangrene" at all, but a diphtheria of the vulva. Yet the symptoms were those of gangrene, the local conditions were identical with those described by authors of much experience as those of hospital gangrene, and were so pronounced by competent surgeons who saw the case, but who did not know of the mode of infection.

So far as I know, this is the only case that I ever infected in my work as a surgeon.

### II.—THE RELATION OF TUBAL PREGNANCY TO PELVIC HEMATOCELE.

There can be no doubt that ectopic pregnancy is much more common than we have been taught to believe. During the past four weeks seven cases of ruptured tubal pregnancy have been reported to the St. Louis Medical Society, with presentation of the specimens removed by abdominal section. One of the specimens was not, however, a true ruptured tubal pregnancy, because the operation was made for pelvic hematocele and the fetus was found still in the tube with its enveloping membranes and tubal walls uninjured, the hemorrhage having occurred from a rent in the tube near its uterine extremity. This was by some regarded as a rare condition of affairs, but I am inclined to think differently. In my own abdominal work I have operated upon quite a number of cases of ectopic pregnancy which have presented the typical history and specimen; in other instances I have made section upon cases termed "pelvic hematocele," yet they have uniformly proven cases of extra-uterine fetation with hemorrhage. From a careful examination of these, as well as numerous cases in the practice of other operators, I cannot but agree with the assertion that nearly all cases of pelvic hematocele are dependent upon extra-uterine pregnancy. Nor is it at all necessary that the tube rupture, in order to produce the trouble—the blood may pour out of the open fimbriated extremity, for most hematoceles are not extraperitoneal—they form as a coagulum, soft, without the form of a "tumor," and easily scooped out; as a definite tumor of more or less organized clot, which is adherent to the peritoneum and leaves the latter rough upon removal of the mass; and as a tumor, consisting of fetus, blood-clot and ovary surrounded by a so-called cyst-wall—really an envelop developed by organization of the clot—which may form with remarkable rapidity. In one of my cases the coagulum filled the whole pelvis and much of the lower abdomen, the "cyst-wall" extending across just a little below the umbilicus and of considerable resistance when the operation was made—ten days after the rupture.

We must not, of course, lose sight of the fact that the tube may rupture in such a way as to cause the formation of extraperitoneal or "broad ligament hematocele" upon the one hand, or to the development of "broad ligament pregnancy" on the other; nor that diffuse hemorrhage into the abdomen may result. In my only fatal case I found a five months' fetus floating in non-coagulated blood up under the liver. But excluding such cases as these, as well as those rare occurrences, pelvic hematocele from ruptured cysts, etc., it does seem to me that the time is now at hand when we can say that almost invariably pelvic hematocele and ruptured tubal pregnancy are synonymous terms.

Furthermore, in spite of the fact that many cases have recovered (temporarily at least) without operative interference, that treatment which is most thoroughly conservative, viewed from every standpoint, is embraced in abdominal section and removal of the abnormal pelvic contents, whether the diagnosis be pelvic hematocele or ruptured tubal pregnancy.

### III.—PERITONEAL ADHESIONS AND THEIR IMPORTANCE.

It seems to me that too little importance has been attached to peritoneal adhesions; not those alone dependent upon abdominal section, but those more frequent bands due to a peritonitis which may have occurred years before the adhesions gave rise to the painful symptoms or to a functional disturbance of the viscera implicated. The pain may be of the dull, continuous, exhausting variety, or it may be quite intense at times, especially when due to the dragging of organs involved in the adhesions or by constriction of the intestine; even perforation, with septic peritonitis, may result from the latter condition, as in one of my patients. When the pain is dependent upon interruption of the passage of intestinal contents, it is frequently very severe, quite like hepatic or renal colic—the typical pain of chronic obstruction of the bowels.

Yet the recognition of such bands is not easy; in fact, it is usually rather difficult, and even by a diagnosis by exclusion, the existence or non-existence of peritoneal adhesions as a cause of pain is often speculative and the surgeon is compelled to acknowledge that he can only determine by an exploratory incision, which is entirely justifiable. Points of import which greatly assist in a correct interpretation of the symptoms are: (1) The history of an attack of peritonitis, either acute or sub-acute or of an abdominal section. (2) The precise location of the pain and its relation to any previous local inflammation. (3) The existence of an appendicitis which has undergone spontaneous "cure." (4) The time when the pain appears in relation to meals. (5) The character of the pain.

Since the tendency of peritoneal adhesions and bands is to become thinner and eventually to disappear, operation is not urgent unless the symptoms are severe or evidences of marked disturbance, like intestinal obstruction, arise. When, however, such manifestations do present themselves the experienced surgeon should not hesitate to open the abdomen, even though he may not be positive in his diagnosis. Even persistent severe pain is sufficient to warrant section for the purpose of breaking up the adhesions. The relief from such treatment will be permanent as a rule.

2312 SALISBURY STREET.

